# CCL22 as an independent prognostic factor in endometrial cancer patients

**DOI:** 10.1016/j.tranon.2024.102116

**Published:** 2024-09-03

**Authors:** Mareike Mannewitz, Thomas Kolben, Carolin Perleberg, Sarah Meister, Laura Hahn, Sophie Mitter, Elisa Schmoeckel, Sven Mahner, Stefanie Corradini, Fabian Trillsch, Mirjana Kessler, Udo Jeschke, Susanne Beyer

**Affiliations:** a Department of Obstetrics and Gynecology, University Hospital, LMU Munich, Munich, Germany; b Center of Integrated Protein Science Munich, Division of Clinical Pharmacology, University Hospital, LMU Munich, Munich, Germany; c Institute of Pathology, TUM, Munich, Germany; d Department of Radiation-Oncology, University Hospital, LMU Munich, Munich, Germany; e Department of Obstetrics and Gynecology, University Hospital Augsburg, Augsburg, Germany

**Keywords:** Endometrial cancer, CCL22, Immune escape, Regulatory T cell, Tumor microenvironment

## Abstract

**Objectives:**

The chemokine CCL22 is recognized for recruiting immunosuppressive regulatory T-cells (Treg) that contribute to disease progression in various tumor entities helping them to evade the host immune response. Our study aims to identify the expressing cell types and to evaluate the prognostic significance of CCL22 secretion and its association with Treg invasion in endometrial cancer (EC), an immunogenic cancer.

**Methods:**

Specimens from 275 patients with EC and 28 healthy controls were screened immunohistochemically for CCL22. Immunofluorescence double-staining for CCL22 and different immune cell markers was performed. In vitro regulation of CCL22-expression was examined in EC cell lines (Ishikawa+, RL95–2) and human PBMCs in coculture settings via qPCR and ELISA.

**Results:**

Elevated CCL22 staining in tumor cells and CCL22-positive M1-macrophages in tumordistant areas were significantly associated with increased overall survival (OS). Conversely, high, secretory-appearing staining in the peritumoral and intratumoral stroma correlated with reduced OS. Although the analysis of the in vitro coculture model of epithelial tumor- and immune cells revealed PBMCs as the primary source of CCL22, we could confirm expression of the chemokine also in the EC epithelial cells.

**Conclusion:**

Our study suggests that CCL22 in EC is associated with OS, dependent on its location and the cell type producing it. Intracellular upregulation and extracellular secretion must be considered separately when investigating CCL22 expressing cell types in EC. These results may provide evidence for CCL22-mediated Treg recruitment in EC as a potential future therapeutic target.

## Highlights

• The impact of the chemokine CCL22 on the outcome of endometrial cancer (EC) patients depends on its localization and producing cell type.
• Positive correlations between the Treg-marker FoxP3 and CCL22 provide hints for CCL22 and Treg as potential targets to overcome immune evasion in EC.
• Intracellular upregulation and extracellular secretion must be considered separately when investigating CCL22 expressing cell types.

## Introduction

Endometrial cancer is the sixth most frequent cancer in women worldwide [1]. Despite low cancer-related death rates, the increasing incidence shows the need for further research on treatment options [2]. The importance of reconsidering diagnosis and therapy is also reflected in the new molecular classification for EC and recent drug approvals for EC immunotherapy [3,4]. However, a more detailed understanding of the tumor microenvironment (TME) is still required to overcome tumor-induced immune escape and the subsequent created immunosuppressive milieu [[5], [6], [7], [8], [9]].

The chemokine CCL22 was originally found to be secreted by macrophages and dendritic cells (DC) [10]. Recently, it has been reported that other immune cells such as monocytes and T-cells as well as epithelial cells and several cancer cells, i.e. breast cancer cells, express CCL22 [11,12,13]. Chemokines bind to chemokine receptors, leading to alterations in cellular adhesion molecules on the target cell and thereby promoting directed migration of immune cells along the chemokine concentration gradient [14]. Consequently, they play a crucial role in mediating the immune response by influencing immune homeostasis within the tumor-stroma microenvironment [15].

CCL22 is a ligand of CCR4 [12], that is preferentially expressed on regulatory T-cells (Treg) and mediates Treg migration into the tumor tissue via chemokine gradient formation [9,12,16]. With Treg being associated with suppression of the local immune response [16], this mechanism may lead to reduced antitumor immunity and favor tumor progression. We recently showed that a higher infiltration of Treg in EC tissue is associated with a reduced OS [17]. A higher expression of CCL22 has been described to be associated with a negative prognostic outcome in several cancers [18,19]. Thus, the recruitment of Treg mediated by CCL22 is an important component of tumor immune escape. Targeting this process could help to strengthen or reinitiate the host immune response against the tumor [16].

Although the role of CCL22 has already been documented in several tumor types, data in EC are missing. This study aimed to elucidate the prognostic value of CCL22 in a large cohort of patients with EC and to evaluate the dynamics of CCL22-expression in-vitro cell culture models of EC. Additionally, we have examined the interplay of CCL22 and Treg, connecting these two markers for the first time in EC.

## Materials and methods

### Patients and specimens

Tissue samples from 275 patients with endometrial adenocarcinoma, treated by surgery in the LMU Munich between 1990 and 2002, were included. Clinicopathological data were retrieved from the Munich cancer registry and complemented by the latest revision of the International Federation of Gynaecology and Obstetrics (FIGO) system from 2009. Information about the histological subtype was reviewed by the Department of Pathology, LMU Munich. Additionally, a control group of 28 specimens from surgeries for benign indications between 2000 and 2002 were enrolled. According to medical records, no evidence for (pre-)malignant or inflammatory processes was found in these samples.

The study was approved by the local ethics committee of the LMU Munich (reference number 19–249). Patients’ data were anonymized. The ethical principles adopted in the Declaration of Helsinki 1975 have been respected. Details for used material see **Supplement 1**.

### Immunohistochemical staining with CCL22

Paraffin-embedded TMA of EC-patients and tissue of the control group were incubated with the polyclonal rabbit anti-human MDC (CCL22) antibody (500-P107 1:300, Peprotech) using ZytoChem Plus HRP Polymer System mouse/rabbit following the manufacturer's instructions. 3,3-diaminobenzidine served as chromogen and slides were counterstained with Meyer`s hemalum. Staining was evaluated by the semiquantitative immunoreactive score (IRS-Score) [20] using a light microscope.

Uterine tissue was divided into the following areas according to histomorphological aspects: endometrial epithelium and areas with stroma and myometrium, depending on the extent of tumor myometrial invasion. Separate scores were collected for epithelial tumor cells of the endometrium and surrounding stroma and myometrium. In addition, the occurrence of conspicuously strong positive isolated cells was noted separately.

### Double immunofluorescence

Primary antibodies (**Supplement 1**) were applied on 27 representative EC-specimens (10 % of the whole cohort) after blocking with Ultra-Vision-Proteinblock. Goat-anti-mouse-Alexa-Fluor488- and Goat-anti-rabbit-Cy-3-conjugated antibodies were used as secondary antibodies. Subsequently, the samples were fixed with Vectashield® H1200 mounting medium with DAPI. Axiophot fluorescent photomicroscope and AxioVision software were used for evaluation. Single-positive and double-positive cells were counted in three representative fields of view (20x magnification) in each section. For CD80-CCL22 staining, serum blocking before applying the primary antibodies was used.

### Cell lines and isolation of primary PBMCs

The human cell lines RL95–2, HEK293 and Ishikawa+ER were maintained in RPMI- 1640 medium + GlutaMAX and 10 % fetal calf serum in a humified incubator at 37 °C with 5 % CO_2_.

Human PBMCs were purified from healthy blood donors by density gradient centrifugation with Biocoll Separating Solution and subsequent erythrolysis.

### Coculture of tumor cells and immune cells

The coculture experiments were performed using transwell inserts to separate the different cell-fractions. HEK293 cell line served as benign control. To establish coculture for an ELISA of supernatants (SN) and qPCR, 2 × 10⁵ tumor cells or HEK293-cells per well were transferred into a 24-well plate. 2 × 10⁶ PBMCs were added in a 0.4-μm-pore Transwell insert for 48 h.

For performing ELISA of intracellular CCL22, coculture was carried out in 6-well plates with 1 × 10^6^ tumor cells and 1 × 10^7^ PBMCs under otherwise identical conditions.

Tumor cells or PBMCs cultured alone were chosen as a control group in each experiment. Equal cell viability after the coculture setting was confirmed by MTT assay for PBMCs Tumor cells after coculture showed equivalent adherence and density in the well plates as the untreated tumor cells suggesting a comparable viability.

### ELISA of supernatants

Collected SN were quantified by Human CCL22/MDC DuoSet ELISA following the protocols supplied by the manufacturer. The standard curve was created using a four-parameter logistic regression (4PL).

### RNA isolation and RT-qPCR

Total RNA was extracted from the cultured cells using the RNeasy Mini Kit. The RNA concentrations were measured photometrically. 0.3–0.5 µg of RNA was transcripted to cDNA using the MMLV Reverse Transcriptase 1-st-Strand cDNA Synthesis kit. cDNA copies were quantified by TaqMan-PCR with Applied Biosystems 7500 Fast Real-Time PCR System and TaqMan Fast Universal PCR Master Mix. TaqMan Gene Expression Assay was used for priming.

Relative expression of CCL22 was calculated by the 2^−ΔΔCt^ formula using β-actin and GAPDH as housekeeping genes.

### ELISA of RIPA-Lysates

To detect intracellular levels of CCL22 an ELISA for RIPA-lysates was established. After 48 h of coculture, cells were lysed in RIPA-Buffer. Protein concentrations were determined with a Bradford assay. RIPA-lysates were diluted 1:10 before applying to the ELISA plate. Besides that, Human CCL22/MDC DuoSet ELISA was carried out as described above. A spike/recovery and linearity assay were performed to validate the purchased ELISA set for probes with 10 % RIPA-solution. The recovery and linearity were in the required range of 80–120 %. The total protein concentration measured in the Bradford assay was used as a factor for calculating the CCL22 concentration measured by ELISA to compensate for random differences in cell number and thus total protein levels.

### Statistics

Statistical analysis and data processing were performed using Excel 2016 and SPSS 26.0.

Survival analysis was performed using the Kaplan-Meier method with the log-rank test. The Cox-regression analysis was used for multivariate analysis. Spearman´s correlation coefficient ρ was used for bivariate associations.

Differences between groups were compared using Kruskal-Wallis-test/Mann-Whitney-U-test. In the case of multiple testing, Bonferroni-Correction was performed. Data are presented as mean ± SEM (Standard error of the mean) or boxplot. *p* ≤ 0.05 was considered as a statistically significant difference.

## Results

### Patients’ characteristics

The clinicopathological characteristics of the 275 analyzed EC patients were previously described (Table 1A) [17]. TMA-spots with insufficient cells were excluded. This resulted in a reduced cohort of 225 patients for statistical analysis of tumor cell-related expressions. The distribution of parameters remained closely similar to the original cohort (Table 1B). Due to the high age at diagnosis (mean 65.02 years), the maximum follow-up was limited to 200 months.

**Table 1:** Demographic and clinical characteristics of the study population with *n* = 275 (A) and *n* = 225 (B).

A			B		
Characteristics	Patient no (*n* = 275)	%	Characteristics	Patient no. (*n* = 225)	%
Age at diagnosis (years)			Age at diagnosis (years)		
<65	140	50.9	<65	109	48.4
>65	135	49.1	>65	116	51.6
Tumorsize pT			Tumorsize pT		
pT1	220	80.0	pT1	175	77.8
pT2	18	6.5	pT2	16	7.1
pT3	32	11.6	pT3	30	13.3
pT4	3	1.1	pT4	3	1.3
Not available	2	0.7	Not available	1	0.4
FIGO			FIGO		
I	212	77.1	I	167	74.2
II	17	6.2	II	15	6.7
III	38	13.8	III	36	16.0
IV	6	2.2	IV	6	2.7
Not available	2	0.7	Not available	1	0.4
Grading			Grade		
G1	162	58.9	G1	128	56.9
G2	89	32.4	G2	77	34.2
G3	24	8.7	G3	20	8.9
Not available	0	0.0	Not available	0	0
Nodal status			Nodal status		
pN0	176	64.0	pN0	142	63.1
pN1	21	7.6	pN1	21	9.3
pNX	78	28.4	pNX	62	27.6
Metastases			Metastases		
pM0	138	50.2	pM0	109	48.4
pM1	5	1.8	pM1	5	2.2
pMX	132	47.3	pMX	111	49.4
Survival			Survival		
Alive	154	56.0	Alive	119	52.9
Died	121	44.0	Died	106	47.1
Not available	0	0.0	Not available	0	0
Progression			Progression		
None	226	82.2	None	180	80.0
At least one	49	17.8	At least one	45	20.0
Not available	0	0.0	Not available	0	0

### Tumor cells and cells in the microenvironment express CCL22

**Fig. 1:**
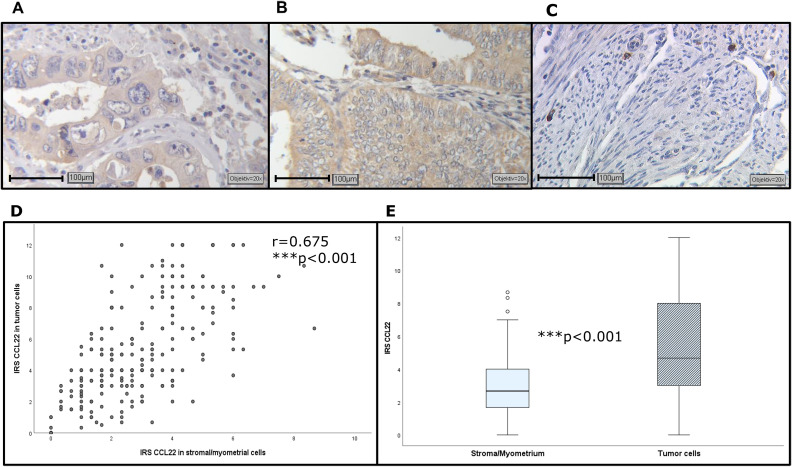
Representative images of CCL22-expression in EC: (A) intermediate expression in glandular cells (IRS=2.67), S/M (IRS=0.67); (B) high expression in glandular cells (IRS=12.00), S/M (IRS=5.33); (C) strongly positive cells in tumor distant S/M; (D) significant correlation between CCL22 in tumor- and stroma-cells; (E) CCL22 levels in tumor cells were higher than in S/M. Objective 20x, Scale bar 100 µm.

**Fig. 2:**
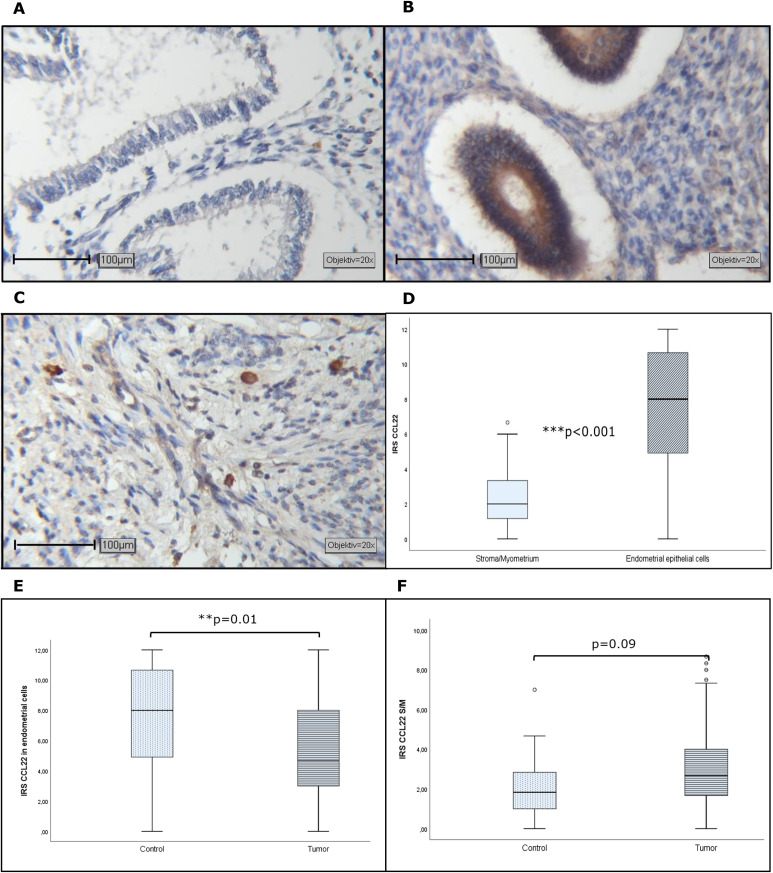
Representative images of CCL22-expression in endometrial control with negative (A) and strong staining in glandular epithelial cells (B) and strongly positive cells in myometrium (C). Elevated CCL22-expression in glandular epithelial cells compared to stroma cells (D). Significantly lower CCL22-IRS EC compared to benign endometrium (E). Concerning CCL22 in S/M a trend to higher levels was found in the specimens of EC patients (F). Objective 20x, Scale bar 100 µm.

Immunohistochemical staining of CCL22 revealed three different areas of expression: 1) the epithelial, endometrial tumor cells showed uniform levels of CCL22 (Fig. 1A); 2) intratumoral and peritumoral stromal or myometrial areas (S/M) were CCL22-positive (CCL22+) without association to specific cells (Fig. 1B). Staining in these areas appeared uniformly pronounced, both intracellularly and extracellularly; 3) single isolated positive cells could be identified in tumor-distant myometrial areas in some cases (Fig. 1C). CCL22-expression in cancer cells was significantly higher than in S/M (Fig. 1D, E).

### CCL22-expression in healthy control group tissue

Analysis of healthy endometrium also revealed that CCL22-expression is present in the different tissue compartments (Fig. 2A-C). Analogous to EC, CCL22-expression in glandular epithelial cells was significantly higher than in surrounding stroma areas (Fig. 2D). Comparison of cancer and healthy tissue revealed a higher glandular epithelial expression in the control group (Fig. 2E) and a trend toward lower expression in the stroma (Fig. 2F).

### High CCL22-expression in endometrial stroma and myometrium is associated with poorer survival

The IRS evaluation of stroma/myometrial areas resulted in a mean value of 2.99 ± 0.11 (range 0.00–8.67).

**Fig. 3:**
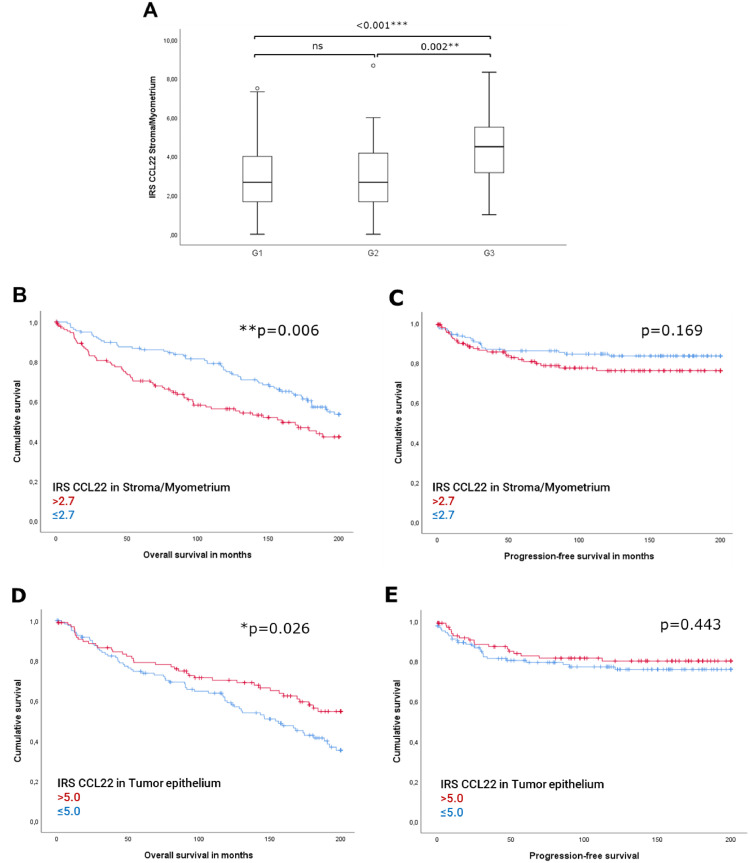
CCL22-expression in S/M increases with higher grade in EC (A). High CCL22-expression in S/M in EC is associated to poorer OS (B), but not to PFS (C). (D) High CCL22-expression in tumor epithelium is associated to prolonged OS, but not to PFS (E).

Advanced disease stages are found to be associated with increasing levels of CCL22-expression and significant differences between the grades (Fig. 3). A significantly positive association between the different levels of grade was found (ρ = 0.175, **p = 0.004). Associations with further clinicopathological parameters are presented in **Supplement 2**.

The median IRS (2.7) was chosen as a cut-off to generate equally distributed patient groups for survival analysis. High expression of CCL22 in stroma/myometrial areas was found to be an unfavorable prognostic factor concerning OS (HR=1.643, CI95 % 1.147–2.353, Fig. 3B), but not progression-free-survival (PFS) (Fig. 3C). Analysis of the estimated 5-year probabilities led to 70.4 %±4 % (mean±SEM) for OS of high-expression group and 86.0 %± 3 % for the low-expression group. According to the latest data, the American Cancer Society reported a 5-year overall survival of 81 % for endometrial carcinoma patients [21].

Identified as a statistically significant predictor in univariate analysis, CCL22-expression in Stroma/Myometrium (S/M) was selected to enter into the Cox-model for multivariate survival analyses. The results revealed CCL22 as an independent predictor of OS (*p = 0.020) after controlling for age, therapy, and pT/FIGO status, but not when additionally adjusting for grade (**Supplement 3**). Due to the high concordance, FIGO and pT staging systems were included separately in the regression model.

### High intracellular CCL22-expression in glandular EC-cells and distant myometrial M1 macrophages is associated to better overall survival

CCL22-expression was detected in almost all EC samples (97 %). The median IRS of CCL22 in EC was 5.3 ± 0.21 (range 0.00–12.00). No significant correlation was found between the expression level of CCL22 in EC and the clinical parameters considered. Survival analysis revealed a significantly better OS for patients with high CCL22-expression in cancer cells (IRS>5; Fig. 3D). Univariate analysis revealed an HR of 0.640 (CI95 %:0.431–0.954). Subsequent multivariate analysis was performed: CCL22 in cancer cells is an independent prognostic factor for a prolonged OS (**Supplement 4**), but not for PFS (Fig. 3E).

**Fig. 4:**
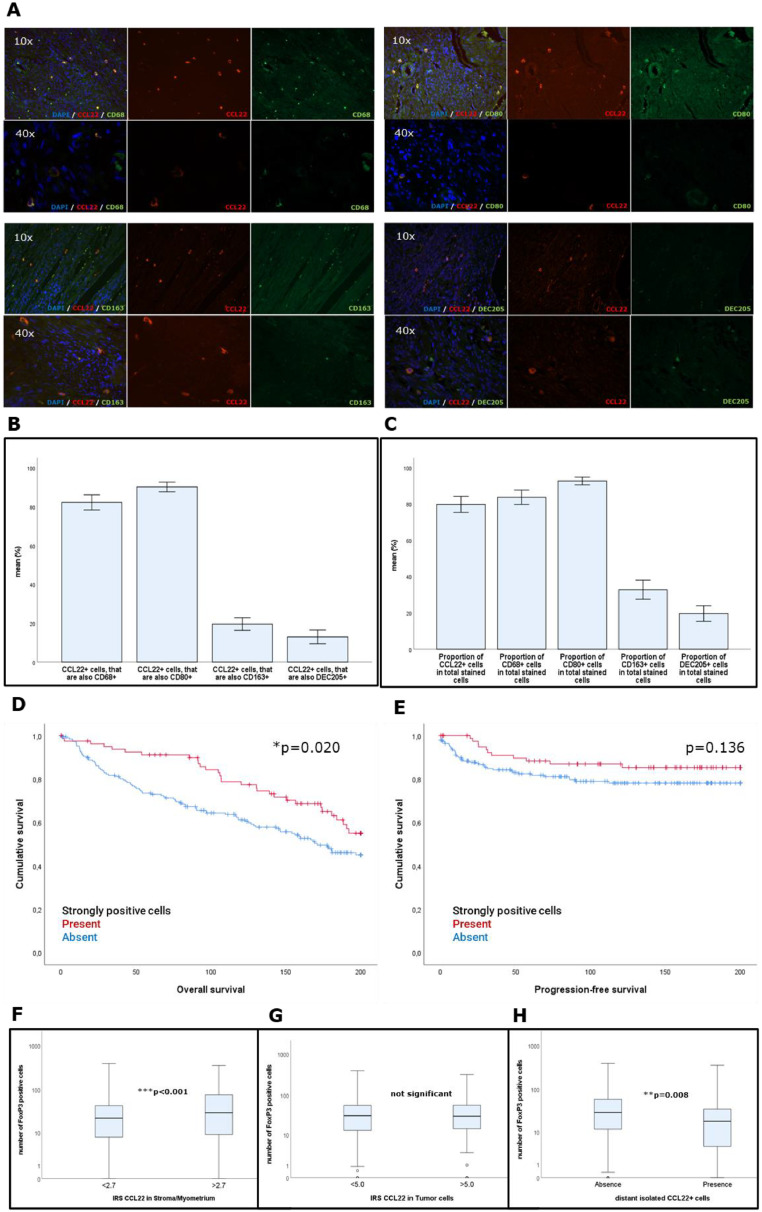
Isolated CCL22+ cells were identified as mainly M1-macrophages in distant myometrial tissue areas by double immunofluorescence: (A) Representative EC stained for CCL22 (red) and CD68, CD80, CD163, and DEC205 (green); (B) Proportion of CCL22+ cells, that expressed also one of those immune cells markers is presented; (C) Proportion of total expression of all markers: dominant occurrence of CD68+ and CD80+ cells; (D) Kaplan-Meier Curve for OS and PFS (E). Correlation analysis of FoxP3 and CCL22 in S/M (F), distant strongly positive cells (G), and in tumor cells (H).

Since isolated strongly CCL22-positive cells were found only in distant myometrial areas (Fig. 1C), they were further characterized by double immunofluorescence (Fig. 4A). We found that the vast majority of these cells are positive for the pan-macrophage marker CD68 and the M1-macrophage marker CD80, while DEC205+ (DC) and CD163 (M2 macrophages) are subordinate coexpressed with CCL22 (Fig. 4B). In conclusion, the distant, strongly CCL22+ cells can be identified as mostly CD68+CD80+ M1-macrophages. Regarding the overall proportion of stained cells, over 80 % of all stained cells were positive for CCL22, CD68, or CD80, respectively. Less than 40 % were positive for CD163 and DEC205 (Fig. 4C).

The presence of those CCL22+ cells was significantly associated with a better OS, (Fig. 4D). Differences in PFS were not significant. A univariate Cox-regression confirmed by the log-rank-test (HR 1.626, CI95 % 1.076–2.459) and adjusting for age, therapy, FIGO/pT, and grade revealed its independence as a prognostic marker (**p = 0.004, HR 1.894, CI95 %:1.233–2.910, **Supplement 5**).

Taken together, we found CCL22 to be expressed by EC cells, distant myometrial M1-macrophages, and in areas of the stroma located close to the tumor. The latter expression appeared intracellular as well as intercellular. The impact on patients’ outcomes depends on the localization of CCL22+ cells.

### Correlation between areas of different CCL22-expression and FoxP3

With CCL22 being the best-known chemoattractant for FoxP3+ Treg, a correlation analysis was performed with FoxP3, which was previously investigated in the same panel. An increased infiltration of Treg was associated with a significantly reduced OS [17]*.* A statistically significant positive correlation was revealed between FoxP3 and CCL22 in S/M (Fig. 4F), but not between FoxP3 and CCL22 expressed by tumor cells (Fig. 4G). Additionally, an association between elevated numbers of FoxP3-positive cells and the absence of distant CCL22+ cells was also found (Fig. 4H).

### Abundant production of CCL22 is a hallmark of non-stimulated PBMCs

Consistent with the existing literature [9,22,23] we detected large amounts of CCL22 by ELISA assay in the SN of freshly isolated PBMCs from healthy humans.

**Fig. 5:**
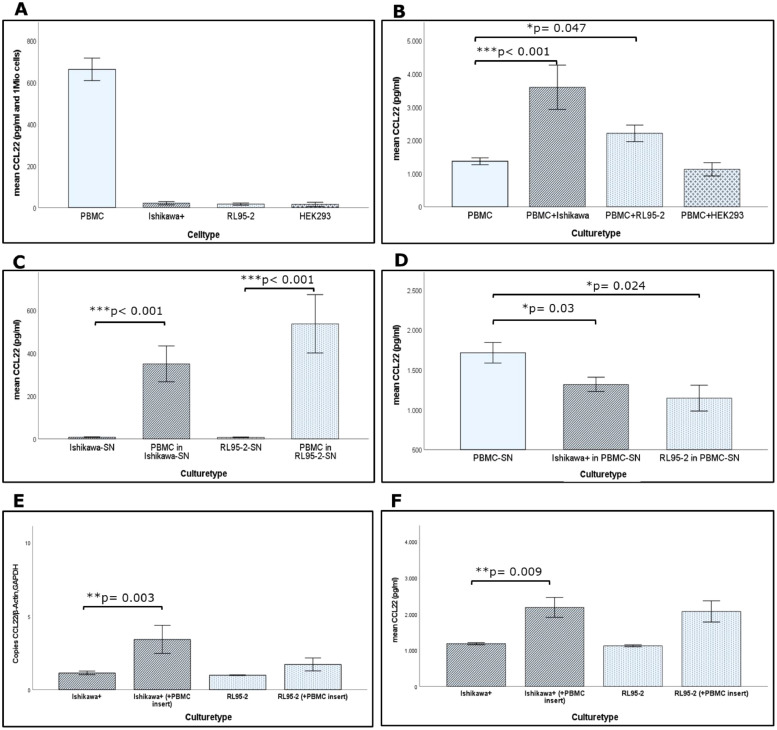
High CCL22 secretion by freshly isolated PBMCs in contrast to EC cell lines without stimulation (A). Significantly increased CCL22 in SN after coculture of PBMCs and EC cell lines (B). The addition of tumor-SN to PBMCs led to a significant increase of CCL22 levels (C), while incubation of tumor cells with PBMC-SN resulted in a decrease in CCL22 levels (D). mRNA levels of tumor cells after coculture revealed a significant increase in CCL22 of Ishikawa+ with PBMCs (E). CCL22 levels of tumor RIPA lysates also revealed a significant increase after coculture (F). All experiments were carried out in technical triplicate and repeated three times with PBMCs from different blood donors.

In contrast to PBMCs, spontaneous CCL22 secretion by the EC-cell lines Ishikawa+ and RL95–2 and the benign control cell line HEK293 was low (Fig. 5A).

### Marginal changes of CCL22 secretion in SN after coculture

To investigate the potential effects of interaction between epithelial tumor cells and immune cells on the CCL22 regulation, coculture experiments were performed.

The addition of PBMCs to the benign cell line HEK293 resulted in a marginally reduced CCL22- concentration in the SN after coculture compared to PBMCs alone (*p* = 0.546). CCL22 levels in the SN of PBMCs cocultured with EC cell lines increased statistically significantly (Fig. 5B). To exclude a further late upregulation of CCL22 secretion, the experiment was repeated after 72 h and 96 h. Levels of CCL22 in the SN of the cocultures decreased below the level of control PBMCs after 72 h and even further after 96 h. This revealed 48 h as the best period to investigate CCL22-induction (**Supplement 6**).

### PBMCs responsible for CCL22 secretion in coculture-SN

To confirm the hypothesis that PBMCs are the main source of CCL22 secretion in coculture-SN, each cell fraction was incubated in cell-free SN of the other cell fraction.

While tumor-SN are devoid of CCL22 secretion, the addition of PBMCs to these SN led to a significant induction of CCL22-production (Fig. 5C), thus suggesting PBMCs are the main source of CCL22.To exclude that, inversely, CCL22 is induced in tumor cells through the SN of PBMCs, we incubated EC cell lines in PBMC culture SN. No upregulation of CCL22 in tumor cells was observed. Culture of tumor cells in cell-free SN of PBMCs resulted in a reduced CCL22 level (Fig. 5D), suggesting either a consumption or uptake of CCL22 by tumor cells or an enhanced degradation of protein in the presence of tumor cells. Therefore, we concluded that CCL22 secretion is restricted to the PBMCs fraction, demonstrating that these cells represent the main source of secreted CCL22 in EC.

### Coculture leads to significantly increased mRNA- and intracellular protein expression in Ishikawa± cells

To elucidate the CCL22-expression on the mRNA level, we performed a quantitative RT-PCR of tumor cells after coculture with PBMCs. When EC cells were prestimulated with PBMCs, CCL22-mRNA-production was markedly enhanced in both EC cell lines, but reached statistical significance only for the Ishikawa+ cell line (Fig. 5E). To confirm our qPCR-data on the protein level, we performed an ELISA analysis of tumor cell lysates after 48 h of coculture with PBMC. The lysates were applied to ELISA plates validated for use with 10 % RIPA solutions to measure the intracellular levels of CCL22. Results showed a significant increase of CCL22 levels expressed by Ishikawa+ cells, whereas the CCL22 level in the RL95–2 coculture did not show a statistically significant increase after coculture with PBMCs (Fig. 5F).

## Discussion

Tumor cells form various mechanisms to create an immunosuppressive microenvironment [24]. The chemokine CCL22 contributes to the recruitment of Treg and is therefore associated with poor prognosis in several types of cancer [13,16,25]. Treg suppress antitumor T-cell immunity and facilitate tumor growth [26]. Therefore, understanding the mechanism of Treg migration and preventing Treg accumulation at the tumor site is essential to overcome immune evasion.

In the present study, we were able to detect CCL22-expression by EC tumor cells as well as in the surrounding TME, and we found correlations for survival prognosis: High CCL22-expression in S/M areas close to EC is associated with poor OS, whereas the presence of CCL22+ M1-macrophages in distant myometrium and the CCL22-expression by tumor cells is linked to a better OS. Generally, the level of expression was higher in tumor cells than in surrounding stroma cells.

The findings from the control group with healthy endometrium are in accordance with these results. Again, CCL22 levels in glandular epithelial cells were significantly higher than in S/M and showed a linear correlation. Comparing the control cohort and EC patients, it was found, that the level in glandular epithelial cells was higher in the control group than in EC cells, while in the stroma, the level was higher in the cancer specimens than in the stroma of healthy endometrium.

Besides EC, there are only a few publications on CCL22 and endometriosis [27] and healthy endometrium [28], which describe an increased CCL22 level in combination with progesterone treatment. An expression in both, stromal and glandular epithelial cells is noted. This suggests an involvement of CCL22 in normal physiology in the balance of local inflammation and immune control via suppression, as observed during pregnancy [28]. Therefore, CCL22-expression is also relevant in healthy endometrium.

Though many studies have revealed the role of CCL22 in Treg accumulation and its impact on OS in cancer, the cellular source of CCL22 and its distribution in cancer tissue is still widely discussed and remains incompletely understood. Some studies describe CCL22 as being exclusively expressed by immune cells like macrophages [29] and DC [22,30], while others report an expression also by tumor cells, like in oral squamous cell carcinoma [31] and breast cancer [9]. Abundant CCL22-expression by tumor cells has been reported following combined crosstalk with NK cells and macrophages via the proinflammatory cytokines IFN-γ, TNF-α and IL1β [9,32,33]. TNF for example is able to induce tissue necrosis and can change the activity of molecules on the cell membrane [34,35]. However, TNF-α IL1β, IL1α, and IFN-α are suggested to play a role in immune cell-derived CCL22-expression [22,30]. In summary, immune cells and tumor cells seem to cooperate in driving the production of CCL22 via proinflammatory cytokines.

Due to the positive correlations of the staining results, CCL22-expression was investigated in cell culture on mRNA, intracellular, and extracellular protein levels in order to trace the cascade of expression at each level. The results of the in vitro cell culture showed a barely measurable basal expression of CCL22 in the EC cell lines. In contrast, a significantly higher level was observed in PBMCs isolated from healthy donor blood. Based on this, we investigated whether the coculture of these cell types would result in an induction of CCL22. This has already been shown for several tumor cell lines [9,22]. We also found in EC an increase of CCL22 in the coculture SN and PBMCs were detected as the relevant secreting cell fraction. However, a closer look at the mRNA and intracellular protein levels in tumor cells revealed also an induction of CCL22 in tumor cells. These results are consistent with histological findings, which showed a particularly high CCL22 level in the tumor cells. It is tempting to speculate that tumor cells might be prevented from secreting CCL22 under certain circumstances, although intracellular expression is increased. Since CCL22 can only exert its effect as a Treg-attracting chemokine extracellularly, impeded secretion results in less Treg invasion and thus reduced tumor progression. Accordingly, CCL22 remaining in tumor cells does not affect Treg invasion and may be associated with a better outcome. But further studies are needed. Specifically, direct cocultures without physical separation could expand the understanding of the importance of cell-cell contact.

PBMCs were also identified as the main producer of CCL22 in the SN in the context of coculture, based on the finding that PBMC incubation with cell-free tumor SN induced a significant upregulation of secreted CCL22. In contrast, the culture of malignant cells in PBMC-SN resulted in reduced levels of CCL22, indicating consumption or uptake by tumor cells or enhanced degradation of the protein. Considering the strong linear correlation of CCL22 between S/M and tumor cells, with higher levels in tumor cells and diffuse staining of the stroma, the possible causes include a mutual stimulation of the cell types and a chemokine uptake by tumor cells from the surrounding stroma.

In contrast to CCL22, FoxP3 has been investigated in some studies on EC, but with contradictory results so far [[36], [37], [38], [39]]. In a previous study, we could show a clear correlation between an increased number of FoxP3+ *T* cells, considered to be Tregs, and a poorer OS [17]. Currently, a strong correlation of CCL22 and FoxP3 in the same cohort was revealed. This is in line with other studies on ovarian and breast cancer [22,40,41] and indicates the potential Treg-recruitment via CCL22. Additionally, the association between elevated numbers of FoxP3-positive cells and the absence of distant CCL22-positive cells could be evidence of an impact of CCL22-expression on FoxP3+ cell infiltration into EC but without necessarily implying direct spatial proximity.

This study reveals the previously unknown prognostic influence of CCL22 in EC and gives insights regarding the expressing cell type. CCL22 is an independent prognostic predictor for OS of EC patients, suggesting its potential as a future target of immunotherapeutic anticancer strategies in EC. While the exact regulation and function of CCL22 in tumor cells needs to be further elucidated, our data suggest that CCL22 levels in stroma and myometrium might be an important parameter for the intratumoral accumulation of immunosuppressive Treg.

## Ethics approval

All procedures performed were in accordance with the ethical standards of the institutional research committee and with the 1964 Helsinki declaration and its amendments. The study was procured in line with WHO Guiding Principles on Human Cell, Tissue and Organ Transplantation.

## Informed consent

The study was approved by the ethics committee of the Ludwig-Maximilians-University Munich (reference number: 048–08; 2008). Patient data were anonymized.

## Consent to participate and consent to publish

not applicable as all data are anonymized.

## Declaration of generative AI in scientific writing

Authors did not use generative artificial intelligence (AI) and AI-assisted technologies in the writing process.

## Funding

This study was supported by “Förderprogramm für Forschung und Lehre” from the LMU Munich (recipient: Thomas Kolben).

## Declaration of generative AI and AI-assisted technologies in the writing process

During the preparation of this work the authors used Grammarly in order to improve language. After using this tool, the authors reviewed and edited the content as needed and take full responsibility for the content of the publication.

## CRediT authorship contribution statement

**Mareike Mannewitz:** Writing – original draft, Visualization, Methodology, Investigation, Formal analysis, Data curation, Conceptualization. **Thomas Kolben:** Writing – review & editing, Supervision, Project administration, Conceptualization. **Carolin Perleberg:** Validation, Supervision, Methodology. **Sarah Meister:** Validation. **Laura Hahn:** Validation. **Sophie Mitter:** Validation. **Elisa Schmoeckel:** Validation. **Sven Mahner:** Resources. **Stefanie Corradini:** Project administration. **Fabian Trillsch:** Project administration. **Mirjana Kessler:** Writing – review & editing, Supervision. **Udo Jeschke:** Supervision, Conceptualization. **Susanne Beyer:** Writing – review & editing, Project administration.

## Declaration of competing interest

The authors declare that they have no known competing financial interests or personal relationships that could have appeared to influence the work reported in this paper.

## Appendix Supplementary materials

Image, application 1

## References

[1] Sung H. (2021). Global cancer statistics 2020: GLOBOCAN estimates of incidence and mortality worldwide for 36 cancers in 185 countries. CA Cancer J. Clin..

[2] Bray F. (2018). Global cancer statistics 2018: GLOBOCAN estimates of incidence and mortality worldwide for 36 cancers in 185 countries. CA Cancer J. Clin..

[3] Kandoth C. (2013). Integrated genomic characterization of endometrial carcinoma. Nature.

[4] Talhouk A. (2015). A clinically applicable molecular-based classification for endometrial cancers. Br. J. Cancer.

[5] de Jong RA (2012). Prognostic role of indoleamine 2,3-dioxygenase in endometrial carcinoma. Gynecol. Oncol..

[6] de Jong RA (2009). Presence of tumor-infiltrating lymphocytes is an independent prognostic factor in type I and II endometrial cancer. Gynecol. Oncol..

[7] Ohno S. (2004). Correlation of histological localization of tumor-associated macrophages with clinicopathological features in endometrial cancer. Anticancer Res..

[8] Schreiber R.D. (2011). Cancer immunoediting: integrating immunity's roles in cancer suppression and promotion. Science.

[9] Faget J. (2011). Early detection of tumor cells by innate immune cells leads to T(reg) recruitment through CCL22 production by tumor cells. Cancer Res..

[10] Godiska R. (1997). Human macrophage-derived chemokine (MDC), a novel chemoattractant for monocytes, monocyte-derived dendritic cells, and natural killer cells. J. Exp. Med..

[11] Mantovani A. (2000). Macrophage-derived chemokine (MDC). J. Leukoc. Biol..

[12] Gobert M. (2009). Regulatory T cells recruited through CCL22/CCR4 are selectively activated in lymphoid infiltrates surrounding primary breast tumors and lead to an adverse clinical outcome. Cancer Res..

[13] Mizukami Y. (2008). CCL17 and CCL22 chemokines within tumor microenvironment are related to accumulation of Foxp3+ regulatory T cells in gastric cancer. Int. J. Cancer.

[14] Johnston B. (2002 Apr). Chemokines in leukocyte adhesion triggering and migration. Semin. Immunol..

[15] et.al Griffith (2014). Chemokines and chemokine receptors: positioning cells for host defense and immunity. Annu. Rev. Immunol..

[16] Curiel T.J. (2004). Specific recruitment of regulatory T cells in ovarian carcinoma fosters immune privilege and predicts reduced survival. Nat. Med..

[17] Kolben T. (2022). Presence of regulatory T-cells in endometrial cancer predicts poorer overall survival and promotes progression of tumor cells. Cell Oncol. (Dordr).

[18] Maolake A. (2017). Tumor-associated macrophages promote prostate cancer migration through activation of the CCL22-CCR4 axis. Oncotarget.

[19] Fu H. (2013). FOXP3 and TLR4 protein expression are correlated in non-small cell lung cancer: implications for tumor progression and escape. Acta Histochem..

[20] Remmele W., Stegner H.E. (1987). Vorschlag zur einheitlichen Definition eines Immunreaktiven Score (IRS) für den immunhistochemischen Ostrogenrezeptor-Nachweis (ER-ICA) im Mammakarzinomgewebe. [Recommendation for uniform definition of an immunoreactive score (IRS) for immunohistochemical estrogen receptor detection (ER-ICA) in breast cancer tissue]. Pathologe.

[21] Siegel R.L. (2021). Cancer statistics, 2021. CA Cancer J. Clin..

[22] Wiedemann G.M. (2016). Cancer cell-derived IL-1α induces CCL22 and the recruitment of regulatory T cells. Oncoimmunology.

[23] Wertel I. (2015). Macrophage-derived chemokine CCL22 and regulatory T cells in ovarian cancer patients. Tumour. Biol..

[24] Zou W. (2005). Immunosuppressive networks in the tumour environment and their therapeutic relevance. Nat. Rev. Cancer.

[25] Maruyama T. (2010). CCL17 and CCL22 chemokines within tumor microenvironment are related to infiltration of regulatory T cells in esophageal squamous cell carcinoma. Dis. Esophagus..

[26] Whiteside T.L. (2014). Regulatory T cell subsets in human cancer: are they regulating for or against tumor progression?. Cancer Immunol. Immunther..

[27] Wang X.-Q. (2017). Synergistic effect of regulatory T cells and proinflammatory cytokines in angiogenesis in the endometriotic milieu. Hum. Reprod..

[28] Jones R.L. (2005). Chemokine expression is dysregulated in the endometrium of women using progestin-only contraceptives and correlates to elevated recruitment of distinct leukocyte populations. Hum. Reprod..

[29] Longoria T.C. (2015). Immunotherapy in endometrial cancer - an evolving therapeutic paradigm. Gynecol. Oncol. Res. Pract..

[30] Rodenburg R.J. (1998). Expression of macrophage-derived chemokine (MDC) mRNA in macrophages is enhanced by interleukin-1beta, tumor necrosis factor alpha, and lipopolysaccharide. J. Leukoc. Biol..

[31] Anz D. (2015). Suppression of intratumoral CCL22 by type i interferon inhibits migration of regulatory T cells and blocks cancer progression. Cancer Res..

[32] Zhao X. (2020). Diminished CD68+ cancer-associated fibroblast subset induces regulatory T-Cell (Treg) infiltration and predicts poor prognosis of oral squamous cell carcinoma patients. Am. J. Pathol..

[33] Nakanishi T. (2006). Expression of macrophage-derived chemokine (MDC)/CCL22 in human lung cancer. Cancer Immunol. Immunther..

[34] Laha D. (2021 Apr 27). The role of tumor necrosis factor in manipulating the immunological response of tumor microenvironment. Front. Immunol..

[35] Wolczyk D. (2016 Aug). TNF-α promotes breast cancer cell migration and enhances the concentration of membrane-associated proteases in lipid rafts. Cell Oncol. (Dordr).

[36] Huang Y.-H. (2019). Cancer-associated fibroblast-derived interleukin-1β activates protumor C-C motif chemokine ligand 22 signaling in head and neck cancer. Cancer Sci..

[37] Guo F. (2020). Tissue infiltrating immune cells as prognostic biomarkers in endometrial cancer: a meta-analysis. Dis. Markers.

[38] Kübler K. (2014). Prognostic significance of tumor-associated macrophages in endometrial adenocarcinoma. Gynecol. Oncol..

[39] Giatromanolaki A. (2008). The presence of tumor-infiltrating FOXP3+ lymphocytes correlates with intratumoral angiogenesis in endometrial cancer. Gynecol. Oncol..

[40] Yamagami W. (2011). Immunofluorescence-detected infiltration of CD4+FOXP3+ regulatory T cells is relevant to the prognosis of patients with endometrial cancer. Int. J. Gynecol. Cancer.

[41] Freier C.P. (2016). FOXP3+ cells recruited by CCL22 into breast cancer correlates with less tumor nodal infiltration. Anticancer Res..

