# Supplementary material for: CCL22 as an independent prognostic factor in endometrial cancer patients
Source: Transl Oncol. 2024 Sep 3;50:102116. doi: 10.1016/j.tranon.2024.102116 (PMC11404215; doi:10.1016/j.tranon.2024.102116)
Supplement: Supplementary file 1 [file mmc1.docx]

| ZytoChem Plus HRP Polymer System mouse/rabbit | Zytomed, Berlin, Germany |
| --- | --- |
| 3,3-diaminobenzidine  Leitz Diaplan lightmicroscope (lens 10x and 20x) | Agilent technologies, Santa Clara, CA, USA  Leica Microsystems, Wetzlar, Germany |
| Ultra-Vision-Proteinblock | Thermo Fisher Scientific, Waltham, MA, USA |
| Mouse monoclonal Anti-CD68 AMAB90874, Dilution 1:800 | Sigma Aldrich, St.Louis, MO, USA |
| Mouse monoclonal Anti-CD163 ab156769, Dilution 1:500 | Abcam, Milton, GB |
| Rabbit polyclonal Anti-CD80 HPA050092, Dilution 1:200 | Sigma Aldrich, St.Louis, MO, USA |
| Mouse monoclonal Anti-DEC205 NCL-L-DEC205, Dilution 1:100 | Novacastra, Nussloch, Germany |
| Rabbit polyclonal Anti-CCL22  500-P107, Dilution IF 1:400, Dilution IHC 1:300 | Peprotech, Rocky Hill, NJ, USA |
| Goat-anti-mouse-Alexa-Fluor488- antibodies | Dianova, Hamburg, Germany |
| Goat-anti-rabbit-Cy-3-conjugated antibodies | Dianova, Hamburg, Germany |
| Vectashield® H1200 mounting medium | VectorLab, Burlingame, CA, USA |
| Axiophot fluorescent photomicroscope | Zeiss, Oberkochen, Germany |
| RL95-2/ HEK293 cell-lines | *The American Type Culture Collection*, Manassas, VA, USA |
| Ishikawa+ER | *European Collection of Cell Cultures*, Porton Down, Salisbury, UK |
| RPMI- 1640 medium + GlutaMAX | Gibco Life technologies, Carlsbad, CA, USA |
| 10% fetal calf serum | Thermo Fischer Scientific, Waltham, MA, USA |
| Biocoll Separating Solution | Biochrom, Darmstadt, Germany |
| Transwell insert | Corning Incorporated, Kennebunk, ME, USA |
| Human CCL22/MDC DuoSet ELISA | Corning Incorporated, Kennebunk, ME, USA |
| RNeasy Mini Kit | Qiagen, Venlo, Netherlands |
| MMLV Reverse Trancriptase 1-st-Strand cDNA kit | Lucigen, Middleton, WI, USA |
| TaqMan-PCR | Thermo Fisher Scientific, Waltham, MA, USA) |
| Biosystems 7500 Fast Real-Time PCR System | Thermo Fisher Scientific, Waltham, MA, USA) |
| TaqMan Fast Universal PCR Master Mix | Thermo Fisher Scientific, Waltham, MA, USA) |
| TaqMan Gene Expression Assay | Thermo Fisher Scientific, Waltham, MA, USA) |
| RIPA-Buffer | Sigma, St. Louis, MO, USA |
| 0.2% protease inhibitor | Sigma, St. Louis, MO, USA |
| Excel 2016 | Microsoft Corporation, Seattle, WA, USA |
| SPSS 26.0 | SPSS, Inc., Chicago, IL, USA |

**Supplement 1.** Details to used materials

|  | median IRS CCL22 Stroma/Myometrium | p-value (Kruskal-Wallis-Test) |
| --- | --- | --- |
| **Tumorsize pT**  pT1  pT2  pT3  pT4 | 2.67  3.14  3.67  2.33 | 0.308 |
| **Figo staging**  I  II  III  IV | 2.67  2.83  3.33  1.83 | 0.276 |
| **Grading**  G1  G2  G3 | 2.67  2.67  4.5 | **0.001***** |
| **Nodal status**  pN0  pN1 | 2.67  2.00 | 0.405 |
| **Metastases**  pM0  pM1 | 2.67  1.33 | **0.037*** |
| **Age at diagnosis**  <65  >65 | 2.83  2.67 | 0..631 |

**Supplement 2.** Comparison with clinicopathologic parameters showed a significant association of CCL22 in S/M with Grading and M-status.

|  | **p** | **Hazard Ratio (95%CI)** |
| --- | --- | --- |
|  | | |
| **A Covariate** |  |  |
| CCL22 Stroma/Myometrium IRS high vs. low | **0.006**** | 1.643 (1.147-2.353) |

| **B Covariate** |  |  |
| --- | --- | --- |
| CCL22 Stroma/Myometrium IRS high vs. low | **0.006**** | 1.655 (1.153-2.376) |
| Age at diagnosis | **<0.001***** | 1.079 (1.058-1.101) |

| **C Covariate** |  |  |
| --- | --- | --- |
| CCL22 Stroma/Myometrium IRS high vs. low | **0.009**** | 1.622 (1.130-2.329) |
| Age at diagnosis | **<0.001***** | 1.079 (1.058-1.103) |
| Therapy  surgery vs. other | 0.051 | 1.432 (0.999-2.052) |

| **D Covariate** |  |  |
| --- | --- | --- |
| CCL22 Stroma/Myometrium IRS high vs. low | **0.020*** | 1.542 (1.070-2.220) |
| Age at diagnosis | **<0.001***** | 1.082 (1.060-1.105) |
| Therapy  surgery vs. other | 0.538 | 1.131 (0.764-1.675) |
| FIGO (Reference FIGO I)  I vs. II  I vs. III/IV | **<0.001*****  0.310  **<0.001** | 1.434 (0.715-2.874)  2.768 (1.731-4.429) |

| **E Covariate** |  |  |
| --- | --- | --- |
| CCL22 Stroma/Myometrium IRS high vs. low | **0.020*** | 1.544 (1.072-2.222) |
| Age at diagnosis | **<0.001***** | 1.080 (1.058-1.103) |
| Therapy  surgery vs. other | 0.493 | 1.145 (0.778-1.686) |
| pT (Reference pT1)  1 vs. 2  1 vs. ¾ | **<0.001*****  0.204  **<0.001***** | 1.536 (0.792-2.981)  3.241 (2.001-5.250) |

| **F Covariate** |  |  |
| --- | --- | --- |
| CCL22 Stroma/Myometrium IRS high vs. low | 0.098 | 1.375 (0.943-2.004) |
| Age at diagnosis | **<0.001***** | 1.082 (1.060-1.105) |
| Therapy  surgery vs. other | 0.757 | 1.065 (0.716-1.584) |
| pT (Reference pT1)  1 vs. 2  1 vs. ¾ | **<0.001*****  0.229  **<0.001***** | 1.514 (0.771-2.973)  3.185 (1.949-5.206) |
| grade (Reference G1)  G1 vs. G2  G1 vs. G3 | **0.003****  0.370  **<0.001***** | 1.215 (0.794-1.857)  2.711 (1.519-4.836) |

**Supplement 3**. Multivariate Cox-Regression confirmed independency of CCL22 expression in S/M as prognostic factor for OS when adjusting for age, therapy and pT/FIGO, but not with grade.

|  | **p** | **Hazard Ratio (95%CI)** |
| --- | --- | --- |
|  | | |
| **A Covariate** |  |  |
| CCL22 Tumor IRS  high vs. low | **0.026*** | 0.640 (0.431-.950) |

| **B Covariate** |  |  |
| --- | --- | --- |
| CCL22 Tumor IRS  high vs. low | **0.028*** | 0.642 (0.432-0.954) |
| Age at diagnosis | **<0.001***** | 1.075 (1.051-1.100) |

| **C Covariate** |  |  |
| --- | --- | --- |
| CCL22 Tumor IRS  high vs. low | **0.028*** | 0.642 (0.432-0.954) |
| Age at diagnosis | **<0.001***** | 1.076 (1.052-1.100) |
| Therapy  surgery vs. other | 0.165 | 1.311 (0.895-1.920) |

| **D Covariate** |  |  |
| --- | --- | --- |
| CCL22 Tumor IRS  high vs. low | **0.005**** | 0.556 (0.369-0.837) |
| Age at diagnosis | **<0.001***** | 1.083 (1.058-1.109) |
| Therapy  surgery vs. other | 0.877 | 1.063 (0.685-1.557) |
| FIGO (Reference FIGO I)  I vs. II  I vs. III/IV | **<0.001*****  0.221  **<0.001***** | 1.723 (0.755-3.367)  3.088 (1.883-5.064) |

| **E Covariate** |  |  |
| --- | --- | --- |
| CCL22 Tumor IRS  high vs. low | **0.009**** | 0.584 (0.390-0.875) |
| Age at diagnosis | **<0.001***** | 1.081 (1.056-1.107) |
| Therapy  surgery vs. other | 0.768 | 1.063 (0.709-1.592) |
| pT (Reference pT1)  1 vs. 2  1 vs. 3/4 | **<0.001*****  0.132  **<0.001***** | 1.723 (0.849-3.501)  3.398 (2.062-5.600) |

| **F Covariate** |  |  |
| --- | --- | --- |
| CCL22 Tumor IRS  high vs. low | **0.001**** | 0.503 (0.331-0.765) |
| Age at diagnosis | **<0.001***** | 1.036 (1.058-1.109) |
| Therapy  surgery vs. other | 0.868 | 1.036 (0.683-1.572) |
| pT (Reference pT1)  1 vs. 2  1 vs. ¾ | **<0.001*****  0.121  **<0.001***** | 1.758 (0.862-3.586)  3.509 (2.113-5.827) |
| grade (Reference G1)  G1 vs. G2  G1 vs. G3 | **<0.001****  0.686  **<0.001***** | 1.102 (0.707-1.718)  3.475 (1.882-5.064) |

**Supplement 4**. Multivariate analysis confirmed independency of intratumoral CCL22 expression as positive prognostic factor for OS.

|  | **p** | **Hazard Ratio (95%CI)** |
| --- | --- | --- |
|  | | |
| **A Covariate** |  |  |
| CCL22 strongly positive present vs. absent | **0.020*** | 1.626 (1.076-2.459) |

| **B Covariate** |  |  |
| --- | --- | --- |
| CCL22 strongly positive present vs. absent | **0.003**** | 1.870 (1.234-2.834) |
| Age at diagnosis | **<0.001***** | 1.083 (1.061-1.105) |

| **C Covariate** |  |  |
| --- | --- | --- |
| CCL22 strongly positive present vs. absent | **0.004**** | 1.853 (1.223-2.807) |
| Age at diagnosis | **<0.001***** | 1.084 (1.061-1.106) |
| Therapy  surgery vs. other | **0.042*** | 1.450 (1.013-2.076) |

| **D Covariate** |  |  |
| --- | --- | --- |
| CCL22 strongly positive present vs. absent | **0.003**** | 1.906 (1.252-2.901) |
| Age at diagnosis | **<0.001***** | 1.088 (1.065-1.112) |
| Therapy  surgery vs. other | 0.436 | 1.165 (0.793-1.711) |
| FIGO (Reference FIGO I)  I vs. II  I vs. III/IV | **<0.001*****  0.330  **<0.001***** | 1.413 (0.705-2.835)  2.943 (1.853-4.672) |

| **E Covariate** |  |  |
| --- | --- | --- |
| CCL22 strongly positive present vs. absent | **0.001**** | 1.999 (1.308-3.053) |
| Age at diagnosis | **<0.001***** | 1.087 (1.064-1.111) |
| Therapy  surgery vs. other | 0.359 | 1.193 (0.818-1.741) |
| pT (Reference pT1)  1 vs. 2  1 vs. ¾ | **<0.001*****  0.132  **<0.001***** | 1.514 (0.779-2.939)  3.529 (2.236-5.770) |

| **F Covariate** |  |  |
| --- | --- | --- |
| CCL22 strongly positive present vs. absent | **0.004**** | 1.894 (1.233-2.910) |
| Age at diagnosis | **<0.001***** | 1.088 (1.065-1.111) |
| Therapy  surgery vs. other | 0.626 | 1.102 (0.746-1.627) |
| pT (Reference pT1)  1 vs. 2  1 vs. ¾ | **<0.001*****  0.242  **<0.001***** | 1.492 (0.764-2.914)  3.463 (2.142-5.598) |
| grade (Reference G1)  G1 vs. G2  G1 vs. G3 | **0.002****  0.245  **<0.001***** | 1.281 (0.844-1.945)  2.730 (1.555-4.792) |

**Supplement 5.** Multivariate Cox-regression revealed the presence of strongly CCL22+ cells as an independent predictor for increased OS


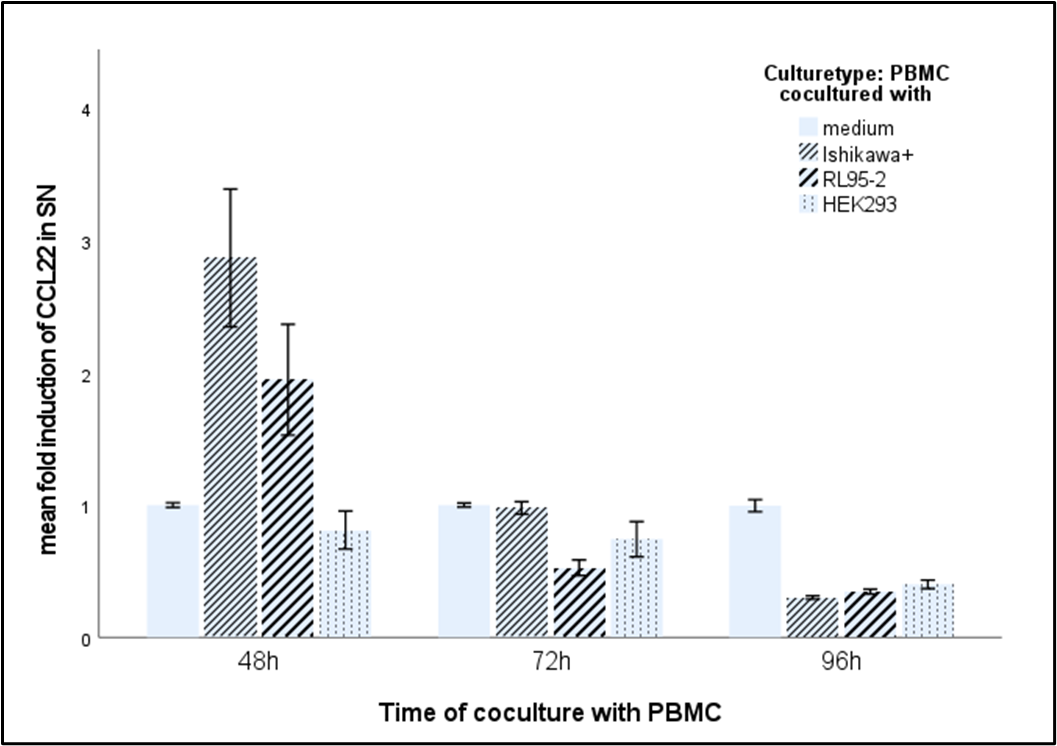


**Supplement 6**. Coculture experiments for CCL22 analysis in culture supernatants were performed after 48, 72 and 96h of coculture. Induction of CCL22 secretion was detected only after 48h in EC cell lines.

All experiments are performed in biological duplicates and technical triplicates.
